# Structural model of corporate social responsibility. An empirical study on Mexican SMEs

**DOI:** 10.1371/journal.pone.0246384

**Published:** 2021-02-16

**Authors:** Martha Ríos-Manríquez, Martha Gabriela Ferrer-Ríos, María Dolores Sánchez-Fernández

**Affiliations:** 1 Department of Finance and Administration, University of Guanajuato, Guanajuato, Mexico; 2 Department of Science and Engineering, Monterrey Institute of Technology and Higher Education, Pachuca de Soto, Mexico; 3 Department of Business, University of A Coruña, A Coruña, Spain; City, University of London, UNITED KINGDOM

## Abstract

Companies are increasingly aware of their role with regard to social responsibility in its three pillars: economic, social and environmental, with their different stakeholders. Facing the dilemma of choosing the model of social responsibility they should adopt, taking care of their organizational culture and their employees, with a global vision that the business world requires. However, it is not an easy task for small and medium enterprises, mainly because of their economic shortcomings in human resources and knowledge of how to be a socially responsible company. But they are aware that Corporate Social Responsibility (CSR) is an opportunity for development and differentiation in the market. Therefore, the objective of this research is to build, identify and validate a model of Social Responsibility in small and medium enterprises in Guanajuato, Mexico (CSRSMEs), on a sample of 226 SMEs, using as a basis the methodology of the international standard of Ethical and Socially Responsible Management System (SGE21). A quantitative approach was used and, a descriptive analysis, exploratory factor analysis and the structural equation modeling was applied. The results determine that the most relevant variables for being socially responsible are human capital, clients, supply chain, social environment and impact on the community, and organizational governance: Legality and Management System. It is drawn from this work that the flexibility of the so-called Ethical and Socially Responsible Management System has the empirical foundations needed. That is, from the perspective of the company’s management to consider the CSRSMEs model an opportunity to adopt and evaluate the areas of social responsibility management of any business structure in the SMEs in Mexico.

## 1. Introduction

Companies of all sizes are becoming increasingly aware of their role with regard to social responsibility in its three pillars: economic, social and environmental, with its different stakeholders being human capital, investors, clients, supply chain, competitors, social factors and the environment [1]. Implementing strategies is affordable for large companies, however, it is not an easy task for Small and Medium Enterprises (SMEs), mainly because of their economic shortcomings in human resources and knowledge of how to be a socially responsible company. Their capacity to adapt to the market [2] and non-bureaucratic and spontaneous performance [3], are qualities which allow them to design strategies to strengthen themselves in the business market. However, Corporate Social Responsibility is an opportunity for growth and differentiation [4] from competitors, as well as to create value for the company and its stakeholders [5].

On the other hand, companies are faced with the dilemma of choosing which model of social responsibility to adopt. In this sense, there are different international proposals such as those issued by the Global Compact, including the International Standard for an Ethical and Socially Responsible Management System (SGE21), which focuses on voluntarily addressing social, environmental and commercial aspects and their relationship with different stakeholders [6]. This standard contemplates nine areas for companies to evaluate their social responsibility: organizational governance, clients, supply chain, human capital, social environment and impact on the community, environmental context, investors, competition and public authorities.

The SGE21 Standard is considered flexible and one of the most complete methodologies for measuring the social responsibility (SR) of any size or type of company. Hence it being considered the basis for this study. The objective of this research is to build, identify and validate a model on Social Responsibility in small and medium enterprises in Guanajuato Mexico (CSRSMEs).

Guanajuato is one of the most important states in the country’s development, ranking sixth in its contribution to the gross domestic product (GDP) in 2017 [7], and in first place for opening a business [8], and therefore converting the state into part of the new industrial heart of Mexico [9], which is why the study was carried out on this state.

This article is structured in six sections. In the introduction, a panorama is established of the reasons that motivated this study, as well as the objectives of this investigation. The second section contains the theoretical framework upon which this study is based, addressing the variables of organizational governance, clients, supply chain, human capital, social environment and impact on the community, environmental context, investors, and competition. This is followed the third section that presents the methodology used in the research follows this. In the fourth section, the results are analysed from the approach of the hypotheses raised, whereas in the fifth section the findings, limitations, future lines and recommendations of the research are laid out. Finally, the references considered in this research are added.

## 2. Literature review

### 2.1. An approach to the concept and expectations of social responsibility in companies

The concept of corporate social responsibiltiy intitally appeared in literature during the second half of the last century [10,11]. However, with the passing of time and its effect on globalization and democratization of the Internet, as well the popularization of social networks and the development of emerging economies, it (corporate social responsibility) became more prominent. Interest in social responsibility (SR) has increased, largely because companies located in developing countries have carried out practices that are unfavourable to workers and the environment. This is why companies which do not engage in these bad practices and wish to differentiate themselves have found in social responsibility the means to do so [10–12]. Through different strategies, companies can satisfy their clients while promoting the development of their collaborators, taking care of the environment and the society in which they are located [10,13]. In this way, social responsibility becomes a factor for a company’s success, expressed through philanthropy, ethical behaviour and a rational use of resources for the benefit of society [10,13–15].

Considering SR as a source of resolution of social problems that are of universal concern [16], it enables the creation of sustainable competitive advantages [4,17–19]. It promotes consumer confidence [20], fulfilling all stakeholders’ expectations [4]. Although it can be manifested through philanthropy [14], it should not be confused with it, since social responsibility has a much broader spectrum, whose actions must go beyond mere legal obligation and promote a true development of society [21] or as a competitive strategy.

The companies face the dilemma of choosing the model of social responsibility they should adopt, considering their organizational culture and their employees, with a global vision that the business world requires. So, international or national organizations are concerned with issuing CSR proposals. Therefore the next section establishes the methodologies, models or instruments that companies can adopt to implement SR.

### 2.2. Models, methodologies, instruments and tools adopted by socially responsible companies issued by international or national organizations

Social responsibility is a management strategy that companies of any size, sector or type are adopting - whether for philanthropic, ethical, stakeholder pressure [22] and/or differentiation purposes, which offers an opportunity to gain competitive advantage [4,23]. SR considers obligations such as workers’ labour rights, legal and environmental requirements that must be fulfilled for the company to work appropriately. They also consider including actions that are adopted voluntarily and go beyond what is strictly obligatory, and even allow them to obtain certification that supports the organization in standardizing its actions in this area and which also allows them to acquire prestige and positioning with reference to other companies [10,24].

There is a great diversity of models, also called methodologies, instruments or tools, which are designed on the basis of the three pillars of social responsibility (SR): economic, social and environmental. These instruments include various indicators proposed by international or national organizations in the country concerned, which enable companies to make the transition to social responsibility. Examples of these instruments include the initiative from the United Nations (UN) through the Global Compact; the Green Book; the Standard AA1000 from the Institute for Social and Ethical Accountability (ISEA) [15]; the guide proposed by international organizations such as the GRI (Global Reporting Initiative) [25]; and the ISO26000 (International Organization for Standardization-Social Responsibility) [26]. In addition, in different countries, there are organizations that propose their own SR certification instruments. For example, in Mexico, CEMEFI (Mexican Center for Philanthropy) [27]; in Spain the Spanish Association for Quality (AEC); Argentina ARSE (Argentine Institutue of Corporate Social Responsibility); in Bolivia, COBORSE (Bolivian Corporation of Corporate Social Responsibility; in Uruguay, DERES (Development of Social Responsibility; Costa Rica, the AED (Association of Entrepreneurs for Development); in Salvador the Fundemas (Business Foundation for Social Action); in Brazil, ETHOS (Ethos Institute for Business and Social Responsibility); and in Guatemala the CENTRARSE (Center for Action for Social Responsibility and Business), among countless countries around the world. Table 1 shows the indicators of models which are considered to integrate the three dimensions of SR and allow for the evaluation of SR performance of any size, sector or type of company in Mexico.

**Table 1:** CSR model indicators: GRI, ISO26000, CEMEFI, AEC, AA1000, SGE21.

International/National Organizations	Indicators
Global Reporting Initiative (GRI) [25].	1) Economic, 2) Social, 3) Environmental.
ISO26000 [26].	1) Governance, 2) Human rights, 3) Labour practices, 4) Environment, 5) Fair operating practices, 6) Consumer issues, and 7) Active participation and community development.
Centro Mexicano para la Filantropía (CEMEFI), (MexicanPhilanthropy Centre), in Mexico, with presence in LatinAmerica [27]	1) Corporate ethics and governance, 2) Quality of life in the company (social dimension of work), 3) Connection and commitment to the community and its development, 4) Care and preservation of the environment.
Asociación Española para la Calidad (AEC) (Spanish Association for Quality) [28].	Company behaviour regarding: 1) Owners, shareholders, investors and partners 2) Company behaviour regardingemployees 3) Clients, users and consumers 4) Supply chain 5) Alliances or partnerships 6) Competitors 7) Administration 8) Community/society 9) Environment
Institute for Social and Ethical Accountability (ISEA), Standard AA1000 [15].	1) Economic, 2) The organization’s social indicators, 3) The way it handles its target audiences.
Forética through the SGE21 Standard [29,30].	1) Organizational governance, 2) Human capital, 3) Clients 4) Supply chain 5) Social environment and impact on the community, 6) Environment context 7) Investors, 8) Competition and 9) Public authorities.

Source: Own elaboration based on literature [15,25–29].

### 2.3. Social responsibility in SMEs in Mexico

As of 2009, the Ministry of Economy in Mexico has established criteria to define the classification of SMEs, taking into account the number of workers, sales and the main orientation of the business. They have established that small enterprises have an annual turnover of between 4.01 and 100 million pesos, when they are in the industrial or service sectors they have between 11 and 50 employees, and when they are in the commercial sector they have between 11 and 30 employees. While the medium-sized enterprises have an annual turnover between 100.01 to 200 million pesos, they have between 31 to 100 employees if they are in the commerce sector; 51 to 100 employees in the service sector and between 51 to 250 employees in the industrial sector.

SMEs are very important to Mexico’s economy [31], and are a fundamental factor in the development of innovation and social promotion [32], as they are one of the main actors in the economy [33]. However, the SMEs have a probability to survive between 6.9 to 9.7 years, and according to the sector, the trading companies survive, on average, 6.9 years, while the service companies 8 years and the manufacturing 9.7 years. In general, 65% of SMEs survive for 5 years on average [34]. SR is an opportunity for growth, differentiation [4,15,22] and customer loyalty. [22]. SR enables a company to improve the environment in which it is located [10,15], but not paying attention to being socially responsible can also negatively influence its profitability [10,35]. For all these reasons, more and more companies are seeking to implement social responsibility strategies, which is practically the new way of doing business. This implies that SMEs voluntarily and through an explicit decision, take responsibility for their activities, both inside and outside the company, and at the same time obtain both economic and image benefits [10].

In Mexico, there have been improvements in the development of the SMEs Social responsibility. But more research is required, based on size of the company, to improve understanding and knowledge of CSR in Mexican SMEs [22].

This research considered the methodology of the International Standard for the Ethical and Socially Responsible Management System (SGE21) of Foretica [6,29], considering it a flexible system with a presence in Europe and Latin America. Delving into the SGE21 Standard below.

### 2.4. Theoretical construction of research indicators and hypotheses

The evolution of business and demand for social commitment from companies, forced such enterprises to go from worrying about maximizing their economic benefits, to the inclusion of social compromise and concern for the environment. Consequently, social responsibility is considered to be the strategy that makes it possible to achieve an optimal condition in which benefits are not only for the organization, beside the stakeholders [36]. Conceptualization can be found in literature under different terms, such as relationship groups or interest groups [27]. In all cases, the equivalent term refers to the individuals to whom the organization’s activities may have an impact on the company’s objectives [10,37].

Social responsibility requires the recognition of stakeholders, as well as generating long-term benefits, both for themselves and for the three areas of SR [10]. To this effect, it is necessary to form a new concept of company based on objectives ranging from trust and pursuit of the common good [11,38] to conscious and honourable interest that promote voluntary integration towards respect, tolerance and the common good [10,20].

The SGE21 standard proposes an ethical and Socially Responsible Management System, composed of nine management areas: 1) organizational governance, 2) clients, 3) supply chain, 4) human capital, 5) social environment and impact on the community, 6) environmental context, 7) investors, 8) competition, 9) public authorities, which companies must establish to demonstrate their social responsibility [6,29,30]. This standard is also flexible and allows for the granting of an CSR certification (corporate social responsibility), after a documentary audit and proving that the company is subject to evaluation on a voluntary basis and is compatible with the SME’s quality, environmental, occupational risk prevention, or innovation management systems [6,29,30].

Considering that the SGE21 Standard (being an international standard) meets the attributes sought in this research, it will raise awareness among SMEs to be socially responsible companies (ESR), with an ethical sense. SGE21 is a global guide, drawn up based on the ISO26000 principles, and is also flexible in a globalized world, with presence in Europe and Latin America. In addition, it has alliances with different organizations at the forefront of SR trends, such as: World Business Council for Sustainable Development (WBCSD), CSR Europe, Business in the Community (BITC), Global Reporting Initiative (GRI), Banco Interamericano de Desarrollo, Global Compact, La Business Social Compliance Initiative (BSCI) and Academy of Business in Society [22]. Based on the Ethical and Socially Responsible Management System of the SGE21 Standard, including the eight management areas in this research: organizational governance, clients, supply chain, human capital, social environment and impact on the community, environmental context, investors, and competitors [29,30], the following hypothesis is proposed:

H_1_. The Social Responsibility of the SME (CSRSMEs) is determined by organizational governance (MS), clients (CL), supply chain (SUP), human capital (HC), the social environment and impact on the community (SE), the environmental context (ES), investors (INV) and competition (COM) of the SMEs in Guanajuato Mexico.

In accordance with the literature, organizational governance have considered the following indicators: management and legality system [39,40]. An adequate management and legal system promotes the solution of structural problems which in turn allows the organization to face increasingly demanding markets, enabling the observance of ethical and environmental standards of companies [39] hence allowing the organization to guide its behaviour through management system [40]. In addition, the SGE21 standard distinguishes that all regulations and legislation are adequately monitored by the organization. The company should also carry out adequate internal management and implement codes of conduct, i.e., decision makers must be governed by a system of accountability management [29]. Management and legality system positively influence a good relationship with the clients [20]. Regarding these statements, the following hypothesis is proposed:

H_2_. The Legality (LEG) influences the Social Responsibility of the SME (CSRSMEs) in Guanajuato Mexico.

The Management system (MS), must have a high degree of awareness and desire to act within a framework of balance amongs society, nature and profitability [4,11,36], so that the organization can fulfill stakeholders’expectations, whilst meeting internal objectives [35], making the company active in sustainable development [11], which is one of the management areas within the SGE21 standard [29,30]. The following hypothesis is therefore proposed:

H_3_. The Management system (MS) influences the Social Responsibility of the SME (CSRSMEs) in Guanajuato Mexico.

The Implementation of SR practices has a positive influence on sales performance [28], so it is important to consider client needs, have good relationships with them and offer quality and responsible information on products and/or services [25]. Clients are essential to a company’s sustainability, which makes them a fundamental area in the SGE21 standard [29,30]. The following hypothesis is therefore proposed:

H_4_. The Clients (CL) influence the Social Responsibility of the SME (CSRSMEs) in Guanajuato Mexico.

The Supply chain are of paramount importance to the organization, as they influence the company’s performance, quality, delivery conditions and prices. These factors affect their SR to such an extent that there is a relationship between the level of SR of companies and their ability to negotiate with supply chain [41,42]. Therefore, they should develop systems to evaluate supply chain and at the same time promote good practices and improvement measures [25], which make it a fundamental area in the SGE21 standard [29,30]. The following hypothesis is therefore proposed:

H_5_. The Supply chain (SUP) influence the Social Responsibility of the SME (CSRSMEs) in Guanajuato Mexico.

Human Capital. Companies must be SR with their employees [36], as it is of the most importance to treat them with dignity, and respect for human rights [6,21,29], to seek their personal welfare, as well as care and monitoring of the working environment [36]. These actions benefit the organization and help increase the satisfaction of its human capital, thereby reducing absenteeism and staff turnover [28] and increasing efficiency and effectiveness. Subsequently the SGE21 standard includes it within its management areas [29,30]. The following hypothesis is therefore proposed:

H_6_. Human capital (HC) influence the Social Responsibility of the SME (CSRSMEs) in Guanajuato Mexico.

Social environment and impact on the community refers to situations that allow society to improve, such as the creation of sources of employment, improvement of employees’ professional development [16], investments that are made in the form of donations and everything that has an impact on the society in which the company operates [32,36], establishing procedures for measuring and evaluating social impact, as well as investment in the community [39], which make it a fundamental area in the SGE21 standard [29,30]. The following hypothesis is therefore proposed:

H_7_. The Social environment and impact on the community (SE) influence the Social Responsibility of the SME (CSRSMEs) in Guanajuato Mexico.

Environmental context. SR has been proposed as a means of safeguarding the environment [35], with the conservation of ecosystems being one of today’s primary concerns and therefore it becomes a social obligation for organizations to respond to the environmental impacts the company may have [40]. Therefore, the organization must identify environmental activities and impacts and establish environmental management programs and strategies to address climate change [25]. Hence the SGE21 standard is concerned about the impact on the environment where companies operate [29,30], presenting the following hypothesis:

H_8_. Environmental context (ES) influence the Social Responsibility of the SME (CSRSMEs) in Guanajuato Mexico.

Investors play a very significant role in the financing, management, control and operation of companies [29,30]. It is important for companies to establish a strategy to guarantee good governance, transparency of information, ownership and management, all aimed at providing investors with clear information [25] (which is appreciated by investors), but not necessarily reflected in the market value of the shares [42], presenting the following hypothesis:

H_9._ The Investors (INV) influence the Social Responsibility of the SME (CSRSMEs) in Guanajuato Mexico.

Companies are responsible for behaving with integrity towards their competitors [29,30]. It suggests that competition should be under fair rules and pricing policies, transparent with a fair use of information without seeking to discourage competition, within a framework of clarity of products and services offered [41]. Actions that the company consider fair competition, cooperation and alliances [39], are a fundamental area in the management system of the SGE21 standard [29,30]. The following hypothesis is therefore proposed:

H_10_. The Competition (COM) influences the Social Responsibility of the SME (CSRSMEs) in Guanajuato Mexico.

This research was addressed to Mexican SMEs, due to their importance in their economy [32], contextualizing in the following section their classification, relevance and the reasons that motivate SMEs to be SR.

## 3. Review empirical method of the investigation

This section presents the hypotheses, sample, a survey instrument, and indicators of the study, in accordance with the proposed objective. Based on all this, a non-experimental study with a quantitative approach was designed using a descriptive analysis, an exploratory factor analysis, a confirmatory factor analysis, and the application of structural equation modeling in order to propose the CSRSMEs model. The statistical package SPSS version 21, STATA 13, SPSS AMOS version 21 was used.

### 3.1. Type of investigation; spatial and temporal scope

This cross-sectional investigation collates data from owners, managers/directors of the human resources department of SMEs in the state of Guanajuato, Mexico between January and December 2018. This research has a quantitative approach using a descriptive analysis, an exploratory factor analysis and a confirmatory factor analysis. A multivariate technique was also applied to structural equation modeling, in which multiple regressions and factor analysis [43] was developed, along with a validity assessment [44]. The sample obtained was made up of 226 SMEs from Guanajuato, Mexico. Measures of the goodness-of-fit, together with incremental adjustment measures and measures to adjust parsimony, were used in order to corroborate the correct adjustment with the empirical data (established in the Table 8), making the SEM a powerful [45] tool and suitable for this research.

### 3.2. Survey instrument, variables and indicators of the social responsibility of SMEs

The survey instrument was adapted to the Mexican context of the management areas of the SGE21 Standard of Forética [29,30], taking into consideration eight indicators with 73 items: organizational governance (22), clients (10), supply chain (4), human capital (15), social environment and impact on the community (5), environmental context (8), investors (3), competition (6) and a control question. All items used a 6-point Likert scale, with the following responses: 1. None of the stipulated values are met; 2. Some values are met; 3. Average degree of implantation; 4. Most values are met; 5. They are completely fulfilled and 6. Does not apply. The Public authorities were left out of this investigation as only one question was taken into consideration (see Table 2).

**Table 2:** Operationalization of CSRSMEs variables and indicators.

Variables	Indicators	Codes/Items
Dependent variable		
SME Social Responsibility		CSRSMEs
*Independent variables*		
SGE21 Standard	Organizational governance	MS / MS1-MS22
	Clients	CL / CL24-CL33
	Supply chain	SUP / SUP34-SUP37
	Human capital	HC / HC38-HC52
	Social environment and impact on the community	SE / SE53-SE57
	Environmental context	ES/ ES58- ES65
	Investors	INV / INV66-INV68
	Competition	COM / COM 69-COM74
*Control variables*		
Company size	Small	From 11 to 50 workers
	Medium	From 50 to 250 workers
*Economic activity*	Industry	*IND*
	Commerce	COM
	Service	SERV
	Agriculture	AGR
Company Type	Family Business	EF
	Company with a single owner	EsD
	Anonymous society	SA

Source: Own elaboration.

In relation to the consent of the participants, they filled out the questionnaire anonymously. A letter was sent to the participants, explaining that the questionnaire was anonymous, and no information, that would allow their identification, is collected individually. It was also guaranteed that the study was not carried out individually, since it is an analysis using statistical techniques that require group data and not individuals or human tests. In this work, a study of social responsibility in small and medium-sized companies in Mexico was elaborated, asking anonymously about the information of the social responsibility of SMEs, not humans.

### 3.3. Technical data of the investigation and characteristics of the sample

The size of the SMEs was limited by the number of employees (between 11 to 250 people and for its annual turnover between 4.01 to 200 million pesos) according to the Official Journal of the Federation [46]. The companies population located in Guanajuato, based with the National Statistical Directory of Economic Units of INEGI, is of 11,608 companies [47]. In this study, the population was delimited to 11,102 companies, since, as a requirement, was considered that the companies had a phone or email contact. A sample of 261 was determined with a sample error of 7% with a 95% confidence level. Through simple random sampling, the surveys were applied and the sample was obtained. However, only 226 were valid questionnaires in which 74.8% small companies and 25.2% medium-sized companies (see Table 3) participated.

**Table 3:** Technical data of the investigation and sample participation by size, type of company and sector.

Sample characterization		%
Geographical scope		Guanajuato, México.
Population		1102 SMEs.
Sample size		261 (95% confidence level and 6% sampling error)
Valid questionnaires		226 SMEs
Final sample in the CSRSMEs structural model		211 (suitable sample size according to Hair, Anderson, Tatham and Black, [48].
Sampling method		Simple random.
Data collection		Questionnaire applied personally to the owners, managers and / or manager of Human Resources.
Company size	Small	74.8%
	Medium	25.2%
Company type	Family Business	31.4%
	Company with a single owner	40.7%
	Anonymous society	27.9%
Sector	Industry	57.1%
	Commerce	13.3%
	Services	28.8%
	Farming	0.9%

Source: Authors.

## 4. Results

In regards to responding to the objective of the research, which is to "build, identify and validate a model on Social Responsibility in small and medium enterprises in Guanajuato, Mexico (CSRSMEs)", this section developed the subsections of Exploratory Factor Analysis, Confirmatory factor analysis, and SEM CSRSMEs model.

### 4.1. Instrument and exploratory factor analysis

On the basis of Standard SGE21 [29,30], the validity and reliability of the scale was verified by carrying out an exploratory factor analysis, taking into account the criteria and tests set out in Table 4.

**Table 4:** Criteria and tests in the factor analysis.

Original Scale	Criteria
Factor load	>500 [49,50]
Correlation matrix	There is a 60% correlation >0,3
KMO	>0,5 [51,52]
Bartlett’s sphericity test	Level of significance (Sig) < 0,05 [53,54]
Communalities	Tendency to 1
Total variance explained	Minimum range (60-80%) [55,56]
Maximum likelihood extraction method	Specification number of factors expected according to the amount of information returned (the result is made explicit by identifying the number of factors) [57–59].
Oblique Rotation Method: Promax.	Suitable for research based on theoretical foundations, allowing factors to be correlated, assuming a conceptual association between the latent variables of the proposed research [58–60]
Alfa Cronbach	Reliable scale > 0,7 [61,62].

Source: Own elaboration based on literature [49–62].

Once the different tests were carried out on the original scale with loads greater than 0.500, the following results were obtained: rotation converged into 8 interactions, 7 components of which were able to reproduce 71.888% of the original variability (100%) of the variance (see Table 5).

**Table 5:** Total variance explained and goodness-of-fit test.

Factor	Initial eigenvalues			Sums of charge extraction squared		
	Total	% of variance	% accumulated	Total	% of variance	% accumulated
1	15.821	47.942	47.942	15.243	46.192	46.192
2	3.824	11.588	59.530	3.321	10.063	56.256
3	1.749	5.301	64.831	1.728	5.236	61.491
4	1.241	3.762	68.592	1.057	3.202	64.694
5	1.111	3.365	71.958	.957	2.900	67.594
6	1.002	3.037	74.995	.841	2.549	70.143
7	.843	2.553	77.548	.576	1.745	71.888
….						
33	.090	.271	100.000			

Source: Authors.

Extraction method: maximum probability.

Using the PEARSON correlation matrix, the factorable items were contrasted and checked with the Coefficient Kaiser-Meyer-Olkin (KMO) [63,64], where KMO = 0.943 > 0.600 is considered a high value [63,65]. Bartlett’s spherical test [54] contrasted the existence of significant correlation between the variables c^2^= 594.985, ρ < 0.01 with a critical level (significance) of 0.000. However, the data obtained indicates that the 6 factors with 33 items are sufficient to determine the SR of the SME eliminating the AFE: environmental context, investors and competition, in addition to 40 items, as can be seen in Table 6.

**Table 6:** Pattern matrix.

Items	Factor					
	1	2	3	4	5	6
MS1 Legal requirements					.909	
MS 2 Specific legal requirements					.740	
MS 3 Legislation and regulations					.540	
MS 10 Responsible GE						.784
MS 11 Responsible RS						.873
MS 13 Compliance with the RS plan	.536					
MS 14 Stakeholders	.873					
MS 15 Classify interest groups	.932					
MS 16 Communication with stakeholders	1.029					
MS 17 Stakeholders information	.852					
MS 18 Public policy against corruption	.663					
MS 19 Internal audits of the ethical management system	.901					
MS 20 Internal audits of the RS system	.954					
MS 21 Review of the ethical management system	.833					
MS 22 Review of the RS system	.645					
MS 23 RS status report	.574					
CL 28 Customer satisfaction		.789				
CL 29 Commercial offer		.691				
SUP 34 Responsible advertising				.638		
SUP 35 Responsible shopping				.664		
SUP 36 Supplier diagnosis				.936		
SUP 37 Supplier Evaluation				.788		
HC 40 Equal opportunities		.514				
HC 42 Collaborative unwanted behaviors		.765				
HC 43 Work-life balance		.894				
HC 44 Security and health		.917				
HC 45 Occupational hazards		.798				
HC 48 Disclosure of the code of conduct		.574				
HC 51 Complaints and suggestions about CSR			.525			
HC 52 Record of problems, solutions and effectiveness			.592			
SE 53 Impact on the community			1.045			
SE 54 Social impacts			.700			
SE 55 Transparency			.714			

Source: Authors.

Extraction method: maximum likelihood. Rotation method: Promax with Kaiser normalization.

a. The rotation has converged in 8 iterations.

Determining the internal consistency of the tool with the Cronbach Alpha test, all factors have a high consistency above α > 0.850 [61,62,66,67]. A robust scale is obtained which allows the evaluation of the social responsibility in the following 6 factors of the small and medium-sized enterprises in Guanajuato: organizational governance; clients; human capital; supply chain; social environment and impact on the community; management and legality system, with 33 observations (see Table 7).

**Table 7:** Criteria and tests in exploratory factor analysis.

Original Scale				Final Scale CSRSMEs			
Factors	Items	Cronbach’s Alpha	Deleted items	Factors	Items	Cronbach’s Alpha	Final Scale
Organizational governance	22	0.957	6 (MS 4, MS 5, MS 6, MS 7, MS 8, MS 9)	Organizational governance	2	0.932	MS10, MS11
Clients	10	0.908	8 (CL24, CL25, CL 26, CL27, CL30, CL31, CL32, CL33)	Clients and human capital	8	0.902	CL8, CL29, HC40, HC 42, HC43, HC44, HC45, HC4
Supply chain	4	0.881		Supply chain	4	0.885	P34, P35, P36, P37
Human capital	15	0.937	7 (HC38, HC39, HC41, HC 46, HC47, HC 49, HC50)	Social environment and impact on the community	5	0.904	HC51, HC52, SE53, SE54, SE55
Social environment and impact on the community	5	0.874	2 (SE56, SE57)	Management system	11	0.953	MS13, MS 14, MS15, MS16, MS17, MS18, MS19, MS20, MS21, MS22, MS23
Environmental context	8	0.935	8 (ES58, ES59, ES60, ES61, ES62, ES63, ES64, ES65)	Legality	3	851	MS1, MS2, MS3
Investors	3	0.701	3 (INV66, INV67, INV68)	Total	33	0.962	
Competition	6	0.830	6 (COM69, COM70 a COM)				
Total	73	0.981	40				

Source: Authors.

### 4.2. Confirmatory factor analysis

This section develops the structural equation modeling (SEM), which is considered the most appropriate technique for a series of separate estimates of simultaneous multiple regression equations [68]. With the results obtained in the AFE, and based on the theoretical model of the Standard SGE21, confirmatory modeling of the scale was carried out using confirmatory factor analysis (CFA) in order to obtain an adequate contrast with the hypotheses of the study [69,70]. Covariance instead of correlations, using the "Mahalanobis (D^2^) distance" criterion for the purification of observations, have been analysed to check if the indicators are equivalent [71]. Once this process has been carried out, those that are farther from the centroid have been removed, which do not add value to the variables of the model [48,72,73]. In addition, the criteria set out in Table 8 have also been applied, using the SPSS AMOS 23 statistical package.

**Table 8:** Criteria and analysis properties.

Sample / Items	Sample 226 items, 33 variables
Scale	Acceptable levels
*Analysis Properties of AMOS Structural Equations*:	
Estimation	Maximum likelihood, Estimate mean and intercepts [69]
Output	Minimization history, Standarized estimates, modification indices, indirect, direct & total effects, Factor score weights, Covariances of estimates, Correlations of estimates, tests for normality and outliers.
*Criteria*:	
Mahalanobis Distance (D^2^)	Probability ≤ 0,001 [68,73]. Eliminate observations furthest from the centroid [74–76].
Factor load on variables	≥0,700 [70,77,78].
Avoid Collinearity problems	Loads ≥ 0,700 [70,77–79].
Goodness-of-fit measures	Normed Chi-squared CMIN/df, < 2 [80]; 2 to 5 [48]. Goodness of Fit Index (GIF), 1.0 perfect adjustment [81]. Mean Quadratic Approach Error (RMSEA), ≤ 0,05[73]; or acceptable value 0,05–0.08 [48]. Expected cross validation index (ECVI), near 1 [73].
Incremental fit measures of the model	Comparative Fit Index (CIF), ≥0,900 [82]. Non-normalized Fit Index or Tucker Lewis index (NNFI / TLI), ≥ 0.900 [70]. Regulated Fit Index (NFI), ≥ 0.900 [83,84].
Parsimony adjustment measures	Parsimony Comparative-Fit Index (PCFI), between 0.500 to 0.700. [70,85] Parsimony Standard Adjustment Index (PNFI), Close to 1 [81]. Akaike Information Criterion (AIC), Small value indicates parsimony [70,86] or close to 0 indicates better fit and greater parsimony [70].

Source: Authors.

**Fig 1:**
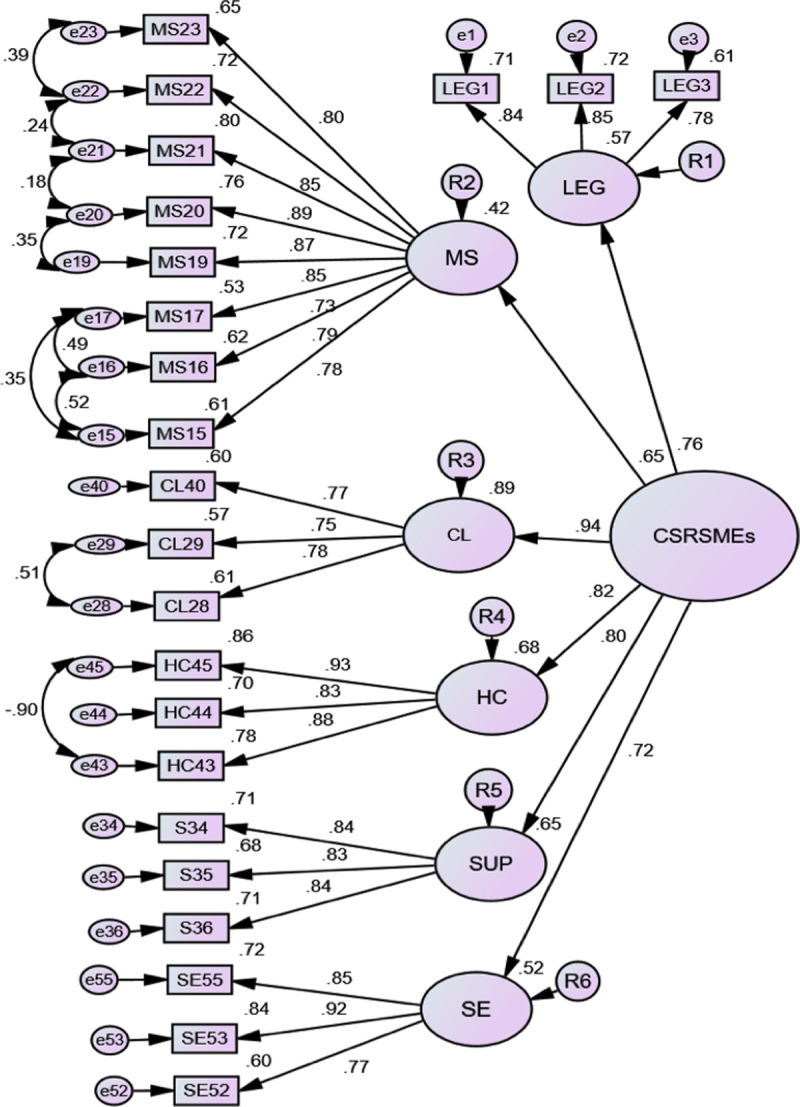
Confirmatory factor analysis of the CSRSMEs model. Notes: Chi-square = 333.900; Probability level=0.000; Degrees of freedom=206; Chi-square normed=1.621. RMSEA = 0.054 LO 90 = 0.043 p=0.243; CFI=0.968; PCFI=0.788; NFI=0.922; PNFI=0.751; TLI=0.961. Source: Own elaboration.

Although the AFE determined a scale of 6 variables with 33 items, the theory for establishing the theoretical model was considered in order to contrast it with the CFA. Therefore they were separated into 2 variables: clients and human capital. The results of the TFA (see Table 9) show the elimination of 14 observations, according to criterion D^2^, for having a probability of 0.001 and 0.000 together with 1 observation having a load of ≤ 0.700. In total 211 observations remain. The latent variable organizational governance was eliminated for not containing "at least 3 observations to avoid problems of identification and convergence" [87, p. 82] (see Fig 1), standardized estimates, loads of observations, variables and errors.

**Table 9:** Statistics of the goodness of fit model: Values obtained.

Absolute fit measures							Incremental or comparative fit measures of the model			Parsimony adjustment measures		
CMIN	P value	DF	CMIN/DF	CFI	RMSEA	ECVI	NFI	TLI	IFI	PCFI	PNFI	AIC
333.900	0.000	206	1.621	0.968	0.054	2.476	0.922	0.961	0.969	0.788	0.751	519.900

Source: Own elaboration.

By analysing the goodness of fit measures of the structural model obtained from the CFA, a better fit was obtained by determining that the normalized chi-square likelihood statistic has an acceptable value of CMIN/DF = 1.621, which reveals a good fit for being less than 2 [80]. To demonstrate the good fit of the model, the Comparative fit index (CIF) was used, which reached a high value of 0.968, indicating a satisfactory fit. In addition, this conforms to the parsimony of the model (PCFI = 0.788) [70,85]. Hence entering an acceptable value in the incremental adjustment rate IFI = 0.969; Standard adjustment index NFI = 0.922, hence a value which indicates that the model improves the fit by 92%.

To be able to overcome the limitations of the NFI in relation to the model of independence, the Tucker Lewis TLI index TLI = 0.961 was used, obtaining a high value [70]. These authors recommend values greater than or equal to 0.947; an average to acceptable value in the standardized parsimony adjustment index PNFI = 0.751. This index relates the observations to the theory which supports them [70]. In summary, the obtained values reveal that the adjustment of the model is appropriate considering the correlations between the errors of measurement of the observations (see Table 9).

It is important to contrast the early adjustment with the population, obtaining an acceptable value in the mean squared error of approximation (RMSEA = 0.054), with a p = 0.243. Consequently, this indicates that the mean squared error of approximation of the model is consistent with reality, that is to say, it represents an anticipated adjustment with the total population beyond the sample [70,73]. With all this in mind, the proposed model fits appropriately [88]. In addition, the correlation between the model variables was observed using indexes to observe the model’s adjustment of structural equation to the model’s parsimony by using the expected cross-validation index ECVI =2.476 and reaching an acceptable value. This index points to an approximation to the goodness of fit that would be obtained with another sample of the same size. Sustained on the fact that, any adjustment result when absolute is indicative that the model is marginally acceptable [89].

Subsequently, it was contrasted that a model prone to over adjustment was not obtained. To be able to contrast a comparative measure between models with dissimilar numbers of constructs, the Akaike AIC Information Criterion = 519.900 was used, obtaining a low value of [70,90] (see Table 9).

### 4.3. SEM CSRSMEs model

**Fig 2:**
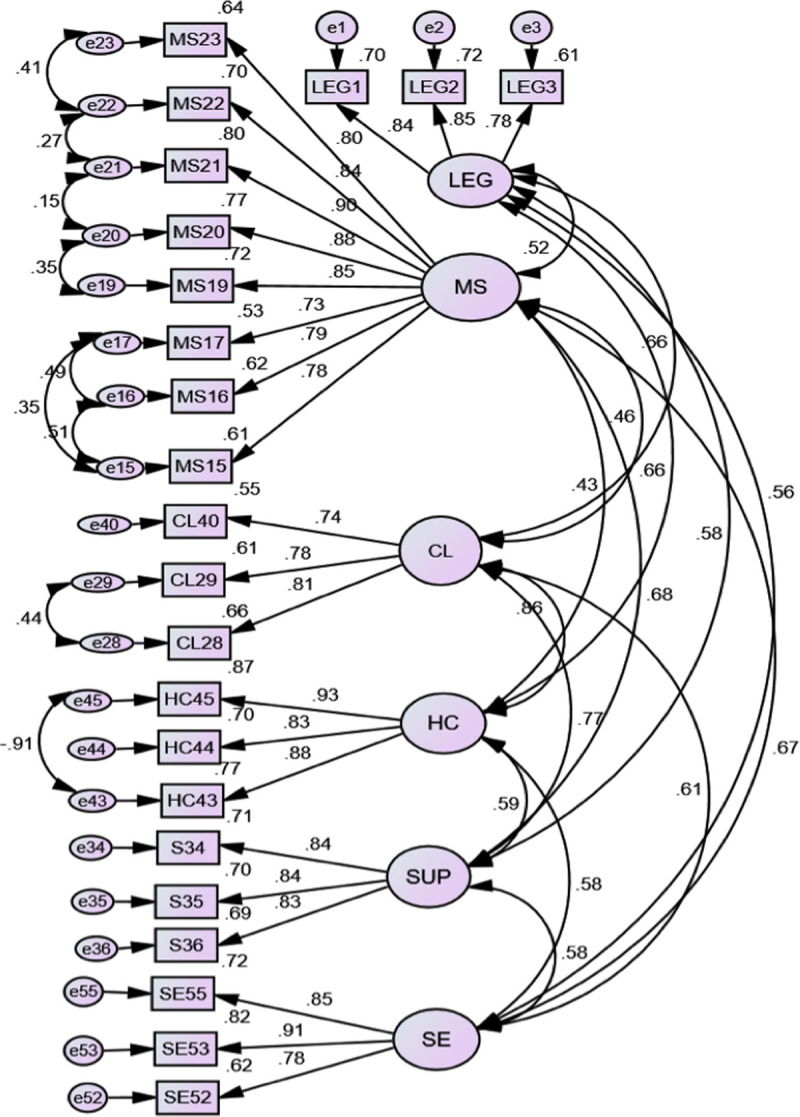
SEM CSRSMEs model. Notes: Chi-square=4222.794; Probability level=0.000; Degrees of freedom=215; Chi-square normed=1.966. RMSEA = 0.068 LO 90=0.58 p=0.001; CFI=0.948; PCFI=0.806; NFI=0.901; PNFI=0.766; TLI=0.939. Source: Own elaboration.

The theoretical basis of this study contemplated 8 dimensions of the SGE21 Standard: 1) management system 2) clients, 3) supply chain, 4) human capital, 5) social environment and impact on the community, 6) environmental context, 7) investors, and 8) competition [6]. Through the AFE and the TFA, the social responsibility (SR) of small enterprises located in Guanajuato, Mexico was established through the application of the structural equation modeling which is based on 6 variables: legality, management system, human capital, clients, supply chain and social environment and impact on the community. Once the standardized estimates were obtained, the loads of observations and variables and errors were presented in Fig 2.

Table 10 shows the internal structure of the CSRSMEs model which has good adjustment rates, CMIN/DF = 1.966; RMSEA = .068; CFI= .948; TLI= .939; NFI = .901; IFI = 0.949; ECVI = 2.813. These results demonstrate that the adjustment of the model is appropriate taking into considertation the correlations between measurement errors in the observations.

**Table 10:** Goodness-of-fit statistic of the CSRSMEs model.

Goodness-of-fit measures	*Acceptability obtained*	*Values*
***Absolute fit measures***		
**Likelihood Statistic (CMIN)**	422.794	Low
**Chi-squared P-value**	0.000	Not acceptable
**CMIN/DF**	1.966	Goodness of fit [80]
Comparatives fit index (CFI)	0.948	High fit [81,91].
**Mean Quadratic Approach Error (RMSEA)**	0.068	The model fits properly [88].
**P-Close**	0.001	Acceptable
**Expected cross validation index (ECVI)**	2.813	Acceptable [89].
***Incremental or comparative fit measures of the model***		
**Regulated index of fit (NFI)**	0.901	Acceptable [70,83,84].
**Non-standard fit index or Tucker Lewis index (NNFI/TLI)**	0.939	Acceptable [70].
**Incremental fit index (IFI)**	0.949	Acceptable [69].
***Parsimony adjustment measures***		
**Parsimony Comparative-Fit Index (PCFI)**	0.806	Conforms to the Parsimony of the model [70,85]
**Parsimony Regulated Fit Index (PNFI)**	0.766	Acceptable [70]
**Akaike information criteria (AIC)**	590.794	Low. Does not indicate parsimony [70,86]

Source: Own elaboration.

In relation to the quality of the adjustment of the structural model, the CSRSMEs model displays an acceptable PCFI parsimony setting, 0.806; PNFI = 0.766. Although it is a low value, in relation to the Information Criterion of Akaike AIC = 590.794, it can be considered that this result does not affect the quality of the CSRSMEs model in an absolute sense. Consequently, based on the results, together with the goodness of fit statistics of the CSRSMEs model, it provides solid evidence of validity.

In order to analyze the reliability, convergent and discriminant validity of the scale, composite reliability (CR) and Variance Extracted (AVE) were used, verifying the convergent validity through β> 0.5 and statistically significant (t-student> + - 1.96), using the following criteria: Average Variance Extracted (AVE)> 0.5, and composite reliability (CR)> 0.7 [48]. Higher values were obtained in each of the following factors: Legality (AVE = 0.824, CR = 0.934); management system (AVE = 0.820, CR = 0.973); clients (AVE = 0.770, CR = 0.909); human capital (AVE = 0.9662, CR = 0.957); supply chain (AVE = 0.837, CR = 0.939); and social environment and impact on the community (AVE = 0.845, CR = 0.942). This demonstrates the convergent and discriminant validity of the model.

## 5. Conclusion, contribution, limitation and future lines of research

Social responsibility is an issue that is currently being implemented in companies, regardless of the motivation to be socially responsible. However, it is undeniable that any type of methodology, be it a national or international standard - such as SGE21, must be adapted. In addition, it should acquire the idiosyncrasies of the country where the company is located and adapt to its culture. This investigation responded to the objective set out, which was the construction, identification and validation of the proposed model, using the Structural Equation Modeling (SEM) on Social Responsibility in small and medium-sized companies in Guanajuato Mexico (CSRSMEs). This by determining the appropriate CRSMEs model to evaluate the CSR of SMEs in Guanajuato, Mexico. Based on the results, together with the goodness of fit statistics of the CSRSMEs model, it provides solid evidence of validity.

The results revealed that CSRSMEs contemplates 6 variables: legality, management system, human capital, clients, supply chain and social environment and impact on the community. The SEM multivariate model was applied, which allowed to establish, according to all the tests carried out, that together with the goodness-of-fit statistics of the CSRSMEs model, a solid evidence of validity was provided.

In the literature, studies were found in Mexico on ISO26000 [92], based on principles from the SGE21 Standard [30,42]. However, only one study of the SGE21 Standard- that is, type of company was conducted using a multiple regression analysis to establish a model, on the Ordinary Least Squares technique [22], establishing that the eight variables of the SGE21 standard are relevant to explain the model. But in this research the determined SEM model consists of six variables: legality, management system, human capital, clients, supply chain and social environment and impact on the community.

In relation to the hypotheses, Hypothesis H_1_ is partially accepted given that "The Social Responsibility of the SME is determined by organizational governance, clients, supply chain, human capital, the social environment and impact on the community, the environmental context, investors and competition of SMEs in Guanajuato Mexico", and since the SGE21 standard considered in the research is of 8 variables, with the CSRSMEs model responding to 6 variables: legality, management system, human capital, clients, supply chain and social environment and impact on the community.

The hypotheses H_2_, H_3_, H_4_, H_5_, H_6_, and H_7_, are accepted because there is a direct, positive and significant relationship between legality, the management system, human capital, clients, supply chain and the social environment and impact on the community. That is, there is a greater dependence or association between the variables, which provide information on their behavior on the path of SMEs towards social responsibility.

Nevertheless, hypothesis H_8_, H_9_, and H_10_ are rejected. These results reveal that SMEs in Guanajuato, Mexico do not prioritize the variables of competition, investors and Environmental context, to be socially responsible. The environmental variable is a very worrying issue since not giving it importance, manifests a lack of responsibility and commitment to sustainability and the care of the environment. In a world with serious problems revolving this issue, action should be taken by all companies. Organizations should establish actions and strategies for environmental care, thus ensuring the well-being of present and future generations.

The value obtained in standarized regression weights, on the latent variables, show a positive and significant connection, impacting on the Social Responsibility of the SME (CSRSMEs). As well as with the values obtained of the RMSEA and pclose, which are acceptable values [88] and the value of Tucker Lewis index= 0.939, which is higher than the recommended minimum [70]. In conclusion, considering the level of error with which this research was carried out, the results confirm the relationship between the dependent variable Social Responsibility of SMEs and the indicators LEG, MS, HC, CL, SUP y SE.

The main contribution of this research was the construction, identification and validation of the CSRSMEs model, which details step by step how a socially responsible model (SR) is established by SMEs located in Guanajuato, Mexico.

The limitation in this research is identified as the sample being small according to some authors [87], who emphazise that results are influenced by the sample size. Although some other authors suggest reaching a minimum value of 200 cases [44,68,93,94]. Considering that this analysis was conducted with 211 observations, it could be seen as a limited sample.

The future lines of research are to expand the sample. It would also be interesting to research models based on differentiation by company size, company type, and business activity or sector. This would allow for the comparison of results. A further proposal would be to expand this research at national level in order to compare different states of Mexico. At international level, this research could be applied in other countries, in order to refute or corroborate the results obtained in this research.

In conclusion, the results determine that for small and medium-sized enterprises, the most relevant dimensions that make up the SGE21 standard to be socially responsible are the human capital, the clients, the supply chain, social environment and impact on the community, legality and management system. This research therefore demonstrates the flexibility of the SGE21 standard [29,30], by confirming that this is an opportunity for the adoption and evaluation of the management areas of any business structure of SMEs in Mexico.

## Supporting information

S1 File (DOCX)

S2 File (DOCX)
