# Supplementary material for: Structural model of corporate social responsibility. An empirical study on Mexican SMEs
Source: PLoS One. 2021 Feb 16;16(2):e0246384. doi: 10.1371/journal.pone.0246384 (PMC7886197; doi:10.1371/journal.pone.0246384)
Supplement: S1 File — (DOCX) [file pone.0246384.s001.docx]

**PROYECTO:**

**“Responsabilidad social empresarial ”**

**CUESTIONARIO SGE21**

Esta encuesta tiene como objeto desarrollar un indicador a nivel empresarial que permita evaluar el nivel de responsabilidad social empresarial desde la perspectiva de la norma SGE21. La información proporcionada por las empresas será tratada bajo estricta confidencialidad. La información agrupada y analizada será proporcionada a las mismas para que sea utilizada como insumo en su toma de decisiones.

**RESPONSABLES DIRECTOS**

Dra. Martha Ríos Manríquez Mtra. Gabriela Ferrer Ríos Dra. María Dolores Sánchez Fernández

[mrm2000mx@gmail.com](mailto:mrm2000mx@gmail.com) gabyfrmx@gmail.com [maria.msflores@gmail.com](mailto:maria.msflores@gmail.com)

Fecha de la encuesta _____ /______ /_____ No. de cuestionario: ­­­­­­**________**

### Sección I.- Datos relacionados de la persona encuestada

| **Hombre_____ Mujer______ Edad____** |
| --- |
| Puesto o cargo que ocupa: Escolaridad: |
| Tiempo en el puesto a su cargo: |

### Sección II.- Datos generales de su empresa

Empresa: Familiar___ con un solo dueño____ Sociedad Anónima____ otra (cuál)________________________

Número de empleados: _____________ Sector: Agricultura____ Industria ____ Comercio _____ Servicios _____

Giro de la empresa: ______________________________________________

Año en que inició operaciones: ____________

Principales líneas de productos o servicios de la empresa:

**CUESTIONARIO SGE21**

**Marque con una X de** 1 a 5 el grado de implantación de las diferentes actividades relacionadas con la responsabilidad social en su empresa:

**1** no se cumplen ninguno de los puntos estipulados.

**2** se cumplen alguno de los puntos.

**3** grado de implantación media.

**4** se cumplen la mayoría de los puntos.

**5** se cumple en su totalidad.

|  | **Alta Dirección** |  |  |  |  |  |
| --- | --- | --- | --- | --- | --- | --- |
|  | **pregunta** | **1** | **2** | **3** | **4** | **5** |
| 1 | Se garantiza que se realiza el seguimiento y control de todos aquellos requisitos legales y normativa que afectan a la actividad de la empresa. |  |  |  |  |  |
| 2 | Se garantiza que se realiza el seguimiento y control de todos aquellos requisitos legales y normativa específica de su sector, entorno local, ambiental, social y laboral. |  |  |  |  |  |
| 3 | Se mantiene actualizada la identificación de la legislación y normativa aplicada. |  |  |  |  |  |
| 4 | Se define y mantiene en la organización una política de gestión ética. |  |  |  |  |  |
| 5 | Se define y mantiene en la organización una política de responsabilidad social. |  |  |  |  |  |
| 6 | Se dispone en la empresa de un código de conducta. |  |  |  |  |  |
| 7 | Se dispone de mecanismos para facilitar la elaboración, revisión, comprensión y cumplimiento del código de conducta. |  |  |  |  |  |
| 8 | Se ha creado un Comité de Gestión Ética. |  |  |  |  |  |
| 9 | Se ha creado un Comité de Responsabilidad Social. |  |  |  |  |  |
| 10 | Se ha nombrado en la empresa un responsable de Gestión Ética. |  |  |  |  |  |
| 11 | Se ha nombrado en la empresa un responsable de Gestión de Responsabilidad Social. |  |  |  |  |  |
| 12 | Se han definido los indicadores de seguimiento correspondientes con el fin de evaluar, al menos anualmente, el cumplimiento del plan. |  |  |  |  |  |
| 13 | Se ha desarrollado un modelo documentado de relación con los grupos de interés. |  |  |  |  |  |
| 14 | Se han establecido los criterios para identificar y clasificar los grupos de interés, así como la metodología para detectar sus expectativas y establecer y priorizar los planes de acción y comunicación. |  |  |  |  |  |
| 15 | Se conservan en la organización evidencias de las comunicaciones con los grupos de interés. |  |  |  |  |  |
| 16 | Se garantiza en la organización la seguridad de la información utilizada y/o conocida de sus grupos de interés desde el inicio de la relación hasta su finalización. |  |  |  |  |  |
| 17 | Se establece en la organización una política pública de lucha contra la corrupción. |  |  |  |  |  |
| 18 | Se realizan en la organización auditorías internas del sistema de gestión ética para comprobar la correcta aplicación del sistema y su adecuación a los requisitos establecidos. |  |  |  |  |  |
| 19 | Se realizan en la organización auditorías internas del sistema de gestión socialmente responsable para comprobar la correcta aplicación del sistema y su adecuación a los requisitos establecidos. |  |  |  |  |  |
| 20 | Se revisa en la organización el sistema de gestión ética al menos anualmente, para asegurarse de su adecuación y eficacia, a través de indicadores de seguimiento y elaboración de planes de mejora continua. |  |  |  |  |  |
| 21 | Se revisa en la organización el sistema socialmente responsable al menos anualmente, para asegurarse de su adecuación y eficacia, a través de indicadores de seguimiento y elaboración de planes de mejora continua. |  |  |  |  |  |
| 21 | Se presenta, al menos cada dos años, un informe de la situación de la responsabilidad social en su organización. |  |  |  |  |  |
|  | **Clientes** |  |  |  |  |  |
| 1 | Se proporcionan productos y servicios responsables y competitivos. |  |  |  |  |  |
| 2 | Se dispone de un compromiso continuo con la investigación, el Desarrollo y la Innovación (I+D+i) [en un documento o carta incluyendo que, para la elaboración del producto o el diseño del servicio se establecen criterios éticos, laborales, sociales y ambientales] |  |  |  |  |  |
| 3 | Los principios de calidad, tanto en la puesta a disposición del producto como en la prestación del servicio forman parte de la cultura organizativa con el objetivo de lograr la máxima satisfacción de los clientes o consumidores. |  |  |  |  |  |
| 4 | Se realizan evaluaciones de satisfacción del cliente, analizando los resultados y poniendo en marcha las medidas de mejora continua. |  |  |  |  |  |
| 5 | Se presenta información clara y honesta de la oferta comercial a los clientes o consumidores. |  |  |  |  |  |
| 6 | Se vela por la seguridad del producto o servicio que pone la organización a disposición de los clientes y consumidores. |  |  |  |  |  |
| 7 | Se provee de accesibilidad global en los productos y servicios suministrados y en las instalaciones mediante la identificación, puesta en marcha y seguimiento de acciones concretas. |  |  |  |  |  |
| 8 | Se establecen principios y prácticas de publicidad responsable conocidos por el Comité de Gestión Ética y Responsabilidad Social y los departamentos afectados. |  |  |  |  |  |
| 9 | Se establece la forma de resolver o de sancionar en caso de incumplir los principios y prácticas de publicidad responsable |  |  |  |  |  |
| 10 | Se aplican los principios y prácticas de publicidad responsable en el proceso de captación comercial. |  |  |  |  |  |
|  | **Proveedores** |  |  |  |  |  |
| 1 | Se han definido los criterios de compra responsable en función de los aspectos éticos, laborales, sociales y ambientales considerados como oportunos y que superan los requisitos legales aplicables. |  |  |  |  |  |
| 2 | Se ha establecido un sistema de diagnóstico y clasificación de los proveedores en función de los distintos niveles de riesgo. |  |  |  |  |  |
| 3 | Se ha establecido una metodología de evaluación de proveedores basada en los criterios de compras responsables previamente definidos que cubre progresivamente a los distintos grupos de riesgos detectados. |  |  |  |  |  |
| 4 | Se colabora con los proveedores en la mejora continua de los resultados obtenidos en el proceso de diagnóstico y evaluación. |  |  |  |  |  |
|  | **Personas que integran la organización** |  |  |  |  |  |
| 1 | Se evidencia, en materia de Derechos Humanos, que se lleva a cabo un control y seguimiento del cumplimiento de los mismos en su relación con el personal de la organización, con atención especial a los ligados a la actividad empresarial. |  |  |  |  |  |
| 2 | Se identifican los distintos perfiles de diversidad de los colaboradores presentes en la organización, priorizando sus expectativas y necesidades y estableciendo planes de acción que garantizan una gestión responsable de dicha diversidad. |  |  |  |  |  |
| 3 | Se garantiza el respeto al Principio de Igualdad de Oportunidades, de forma específica en el acceso a los puestos de trabajo, la formación, el desarrollo profesional y la retribución. |  |  |  |  |  |
| 4 | Se garantiza la ausencia de discriminación por circunstancias de género, origen racial o étnico, religión o convicciones, discapacidad, edad u orientación sexual o cultura, entre otras. |  |  |  |  |  |
| 5 | Se controla expresamente que el personal de la organización no es destinatario de conductas no deseadas que tengan como objetivo o consecuencia el atentado contra la dignidad o la creación de un entorno intimidatorio, humillante u ofensivo. |  |  |  |  |  |
| 6 | Se facilita la conciliación de la vida personal, familiar y laboral de las personas que la integran mediante políticas activas de las cuales se mantienen registros de sus resultados. |  |  |  |  |  |
| 7 | Se desarrollan mecanismos que garantizan la seguridad y salud en todas las áreas de gestión y actividad de la empresa. |  |  |  |  |  |
| 8 | Se garantiza el cumplimiento de las disposiciones en materia de prevención de riesgos laborales. |  |  |  |  |  |
| 9 | Se mantiene actualizado y a disposición de las personas que trabajan en la organización la descripción de los puestos de trabajo (requisitos del puesto, responsabilidades, dependencias jerárquicas y funcionales así como los sistemas y parámetros de evaluación del desempeño). |  |  |  |  |  |
| 10 | Se evalúan periódicamente las necesidades de formación, estableciendo los programas necesarios para que los empleados actualicen y desarrollen sus competencias, de acuerdo con los objetivos generales de la organización. |  |  |  |  |  |
| 11 | Se asegura que los empleados reciban información acerca del Código de Conducta así como de aquellos aspectos relacionados con el sistema de gestión ética y socialmente responsable que inciden en sus áreas de responsabilidad o que son de interés general. |  |  |  |  |  |
| 12 | Se lleva a cabo una evaluación del clima laboral al menos cada tres años, analizando y poniendo en marcha los mecanismos necesarios para su mejora continua. |  |  |  |  |  |
| 13 | Se tienen en consideración, en caso de reestructuración, las necesidades, intereses y demandas de las partes afectadas por el proceso, reduciendo en la medida de lo posible los impactos negativos asociados. |  |  |  |  |  |
| 14 | Se dispone a todas las personas de las vías adecuadas que integran la organización, para dirigir sus sugerencias, quejas o denuncias sobre aspectos relacionados con la Gestión Ética y Socialmente Responsable de la organización. |  |  |  |  |  |
| 15 | Se mantiene un registro de las entradas y las medidas puestas en marcha para resolver las problemática, así como de su eficacia. |  |  |  |  |  |
|  | **Entorno social** |  |  |  |  |  |
| 1 | Se tienen en cuenta las repercusiones que tienen las actividades de la organización en las comunidades en las que opera. |  |  |  |  |  |
| 2 | Se identifican los impactos sociales, tanto positivos como negativos, de la actividad de la organización y se adoptan las medidas oportunas para mejorar la contribución a la sociedad. |  |  |  |  |  |
| 3 | Se vela por la transparencia en la actividad de la organización con respecto a su entorno social, facilitando los cauces de comunicación y cooperación con los grupos de interés. |  |  |  |  |  |
| 4 | Se elabora anualmente un informe de las acciones solidarias o de acción social que se realizan en la organización. |  |  |  |  |  |
| 5 | En dicho informe se incluyen los resultados obtenidos de acuerdo con la importancia social o económica de las mismas. |  |  |  |  |  |
|  | **Entorno ambiental** |  |  |  |  |  |
| 1 | Se compromete la organización de forma púbica a prevenir la contaminación generada por las operaciones y productos, incluyendo estrategias contra el cambio climático, así como mejorar de forma continua su desempeño ambiental favoreciendo el objetivo global de desarrollo sostenible. |  |  |  |  |  |
| 2 | Se identifica, registra y evalúa aquellos aspectos de las actividades desarrolladas por la organización, sus productos y servicios que causan o pueden causar impactos al medio ambiente. |  |  |  |  |  |
| 3 | Se establece un programa de gestión con objetivos y metas medibles y coherentes con el compromiso ambiental para las plantas o centros de trabajo que disponga la empresa, con el objetivo de mejorar los impactos en el entorno producidos por sus actividades. |  |  |  |  |  |
| 4 | Se revisa anualmente el programa de gestión (mencionado en el 6 c1) y siempre que se produzcan cambios en la organización que afectan a la identificación vigente. |  |  |  |  |  |
| 5 | Se dispone de un plan de riesgos para evaluar, prevenir y gestionar los riesgos ambientales asociados a la actividad de la organización, así como mitigar los impactos adversos en el entorno. |  |  |  |  |  |
| 6 | Se incluye en el plan de riesgos los registros de casos de accidentes, incidentes y situaciones de emergencia, así como las medidas tomadas para su corrección y prevención. |  |  |  |  |  |
| 7 | Se revisa anualmente el plan de riesgos anualmente y siempre que se producen cambios en la organización que puedan afectar a la identificación vigente. |  |  |  |  |  |
| 8 | Se informa a todos los stakeholders, al menos cada dos años, sobre aspectos ambientales asociados a la actividad de la organización. |  |  |  |  |  |
|  | **Inversores** |  |  |  |  |  |
| 1 | Se siguen los principios rectores de transparencia, lealtad , y creación de valor de forma sostenible en relación con la organización. |  |  |  |  |  |
| 2 | Se dispone de un protocolo de relaciones con inversores o código de buen gobierno en la organización y también está a disposición de los inversores. |  |  |  |  |  |
| 3 | Se hacen públicas y accesibles las cuentas anuales de la empresa. |  |  |  |  |  |
|  | **Competencia** |  |  |  |  |  |
| 1 | Se respetan los derechos de propiedad de los competidores de la empresa. |  |  |  |  |  |
| 2 | Se fomenta acudir a acuerdos entre las partes o fórmulas de arbitraje, como vía de resolución de diferencias al respecto. |  |  |  |  |  |
| 3 | No se utilizan las acciones indebidas para recabar información sobre los competidores de la organización. |  |  |  |  |  |
| 4 | Se mantiene un registro actualizado que recaba las denuncias y requerimientos realizados por los competidores. |  |  |  |  |  |
| 5 | No se difunde información falseada o tendenciosa en contra los competidores de la empresa. |  |  |  |  |  |
| 6 | Se fomenta la incorporación a asociaciones y foros de interés común, que sirve de encuentro con los competidores de la empresa y de intercambio de experiencias entre los mismos. |  |  |  |  |  |
|  | **Administraciones Públicas** |  |  |  |  |  |
| 1 | Se establecen los canales de comunicación y diálogo oportunos con las Administraciones con las que se relaciona la empresa. |  |  |  |  |  |

**¡ NUESTRO AGRADECIMIENTO POR SU COLABORACIÓN!**
