# Supplementary material for: Structural model of corporate social responsibility. An empirical study on Mexican SMEs
Source: PLoS One. 2021 Feb 16;16(2):e0246384. doi: 10.1371/journal.pone.0246384 (PMC7886197; doi:10.1371/journal.pone.0246384)
Supplement: S2 File — (DOCX) [file pone.0246384.s002.docx]

**PROYECTO:**

**“Corporate Social Responsibility”**

**Questionnaire SGE21**

The purpose of this survey is to develop an indicator at the corporate level that will allow for the evaluation of the level of corporate social responsibility from the perspective of the SGE21 standard. The information provided by the companies will be treated under strict confidentiality. The information gathered and analyzed will be provided to the companies so that it can be used as input in their decision making.

**DIRECT SUPERIORS**

Martha Ríos Manríquez Gabriela Ferrer Ríos María Dolores Sánchez Fernández

martha@ugto.mx; mrm2018mx@gmail.com gabyfrmx@gmail.com maria.msflores@gmail.com

Date of survey _____ /______ /_____ Questionnaire No: ­­­­­­**________**

### Section I.- Related data of the respondent

| **Male_____ Female______ Age____** |
| --- |
| Position or position held: Schooling: |
| Time in your position:: |

**Section II - General information about your company**

Company: Family___ with only one owner____ Sociedad Anónima____ another (which one)____________________

Number of employees: _____________ Sector: Agriculture ____ Industry ____ Trade _____ Services _____

Company's business: ______________________________________________

Year in which it began operations: ____________

Main lines of products or services of the company:

**Questionnaire SGE21**

**Mark with an X from 1 to 5 the degree of implementation of the different activities related to social responsibility in your company:**

**1** none of the stipulated points are fulfilled.

**2** any of the points are fulfilled

**3** average degree of implementation.

**4** most of the points are fulfilled.

**5** is fulfilled in its totality.

|  | **Senior Management** |  |  |  |  |  |
| --- | --- | --- | --- | --- | --- | --- |
|  | **ask** | **1** | **2** | **3** | **4** | **5** |
| 1 | It is guaranteed that the monitoring and control of all those legal requirements and regulations that affect the company's activity is carried out. |  |  |  |  |  |
| 2 | It is guaranteed that the monitoring and control of all those legal requirements and specific regulations of its sector, local environment, environmental, social and labor. |  |  |  |  |  |
| 3 | The identification of the legislation and regulations applied is kept up to date. |  |  |  |  |  |
| 4 | An ethical management policy is defined and maintained in the organization. |  |  |  |  |  |
| 5 | A policy of social responsibility is defined and maintained in the organization. |  |  |  |  |  |
| 6 | A code of conduct is available in the company. |  |  |  |  |  |
| 7 | Mechanisms are in place to facilitate the development, review, understanding and compliance with the code of conduct. |  |  |  |  |  |
| 8 | An Ethical Management Committee has been created. |  |  |  |  |  |
| 9 | A Social Responsibility Committee has been created. |  |  |  |  |  |
| 10 | A person in charge of Ethical Management has been appointed in the company. |  |  |  |  |  |
| 11 | A person responsible for Social Responsibility Management has been appointed in the company. |  |  |  |  |  |
| 12 | The corresponding monitoring indicators have been defined in order to evaluate, at least annually, the fulfilment of the plan. |  |  |  |  |  |
| 13 | A documented model of relationship with stakeholders has been developed. |  |  |  |  |  |
| 14 | Criteria have been established to identify and classify interest groups, as well as the methodology to detect their expectations and to establish and prioritize action and communication plans. |  |  |  |  |  |
| 15 | Evidence of communications with stakeholders is kept in the organization. |  |  |  |  |  |
| 16 | The security of the information used and/or known about the stakeholders is guaranteed in the organization from the beginning of the relationship until its end. |  |  |  |  |  |
| 17 | A public policy against corruption is established in the organization. |  |  |  |  |  |
| 18 | Internal audits of the ethical management system are carried out in the organization to verify the correct application of the system and its adaptation to the established requirements. |  |  |  |  |  |
| 19 | Internal audits of the socially responsible management system are carried out in the organization to verify the correct application of the system and its adaptation to the established requirements. |  |  |  |  |  |
| 20 | The ethical management system is reviewed in the organization at least annually, to ensure its adequacy and effectiveness, through monitoring indicators and development of continuous improvement plans. |  |  |  |  |  |
| 21 | The socially responsible system is reviewed in the organization at least annually, to ensure its adequacy and effectiveness, through monitoring indicators and development of continuous improvement plans. |  |  |  |  |  |
| 21 | A report on the status of social responsibility in your organization is presented at least every two years. |  |  |  |  |  |
|  | **Clients** |  |  |  |  |  |
| 1 | Responsible and competitive products and services are provided. |  |  |  |  |  |
| 2 | There is a continuous commitment to research, development and innovation (R+D+i) [in a document or letter including that, for the elaboration of the product or the design of the service, ethical, labor, social and environmental criteria are established]. |  |  |  |  |  |
| 3 | The principles of quality, both in the provision of the product and the service, are part of the organizational culture with the aim of achieving maximum satisfaction of customers or consumers. |  |  |  |  |  |
| 4 | Customer satisfaction evaluations are carried out, analysing the results and implementing continuous improvement measures. |  |  |  |  |  |
| 5 | Clear and honest information of the commercial offer is presented to the clients or consumers. |  |  |  |  |  |
| 6 | The safety of the product or service that the organization makes available to the clients and consumers is guaranteed. |  |  |  |  |  |
| 7 | Global accessibility is provided in the products and services supplied and in the facilities by means of the identification, implementation and monitoring of specific actions. |  |  |  |  |  |
| 8 | Principles and practices of responsible advertising are established and known by the Ethical Management and Social Responsibility Committee and the departments concerned. |  |  |  |  |  |
| 9 | The way to solve or sanction in case of noncompliance with the principles and practices of responsible advertising is established |  |  |  |  |  |
| 10 | The principles and practices of responsible advertising are applied in the process of commercial recruitment. |  |  |  |  |  |
|  | **Suppliers** |  |  |  |  |  |
| 1 | Responsible purchasing criteria have been defined based on ethical, labor, social and environmental aspects considered appropriate and that exceed applicable legal requirements. |  |  |  |  |  |
| 2 | A system of diagnosis and classification of suppliers has been established according to the different levels of risk. |  |  |  |  |  |
| 3 | A supplier evaluation methodology has been established based on previously defined responsible purchasing criteria that progressively covers the different groups of risks detected. |  |  |  |  |  |
| 4 | We collaborate with suppliers in the continuous improvement of the results obtained in the diagnosis and evaluation process. |  |  |  |  |  |
|  | **People within the organisation** |  |  |  |  |  |
| 1 | In terms of human rights, it is evident that a control and monitoring of compliance with them is carried out in their relationship with the organization's personnel, with special attention to those linked to business activity. |  |  |  |  |  |
| 2 | The different diversity profiles of the collaborators present in the organization are identified, prioritizing their expectations and needs and establishing action plans that guarantee a responsible management of such diversity. |  |  |  |  |  |
| 3 | Respect for the Principle of Equal Opportunities is guaranteed, specifically in access to jobs, training, professional development and remuneration. |  |  |  |  |  |
| 4 | The absence of discrimination due to gender, racial or ethnic origin, religion or beliefs, disability, age or sexual orientation or culture, among others, is guaranteed. |  |  |  |  |  |
| 5 | It is expressly controlled that the organization's personnel is not the target of undesired conduct that has the objective or consequence of violating dignity or creating an intimidating, humiliating or offensive environment. |  |  |  |  |  |
| 6 | The conciliation of personal, family and work life of the people who integrate it is facilitated by means of active policies of which records of their results are kept. |  |  |  |  |  |
| 7 | Mechanisms are developed to guarantee health and safety in all areas of management and activity of the company. |  |  |  |  |  |
| 8 | Compliance with occupational risk prevention regulations is guaranteed. |  |  |  |  |  |
| 9 | The description of the jobs (job requirements, responsibilities, hierarchical and functional dependencies as well as the systems and parameters of performance evaluation) is kept updated and available to the people working in the organization. |  |  |  |  |  |
| 10 | Training needs are periodically evaluated, establishing the necessary programs for employees to update and develop their skills, in accordance with the general objectives of the organization. |  |  |  |  |  |
| 11 | It is ensured that employees receive information about the Code of Conduct as well as those aspects related to the ethical and socially responsible management system that affect their areas of responsibility or are of general interest. |  |  |  |  |  |
| 12 | An evaluation of the work climate is carried out at least every three years, analysing and implementing the necessary mechanisms for its continuous improvement. |  |  |  |  |  |
| 13 | In case of restructuring, the needs, interests and demands of the parties affected by the process are taken into consideration, reducing as much as possible the associated negative impacts. |  |  |  |  |  |
| 14 | All the people who make up the organization are provided with the appropriate channels to address their suggestions, complaints or claims on aspects related to the Ethical and Socially Responsible Management of the organization. |  |  |  |  |  |
| 15 | A record is kept of the entries and the measures put in place to resolve the problems, as well as their effectiveness. |  |  |  |  |  |
|  | **Social environment** |  |  |  |  |  |
| 1 | The impact of the organization's activities on the communities in which it operates is taken into account. |  |  |  |  |  |
| 2 | The social impacts, both positive and negative, of the organization's activity are identified and appropriate measures are taken to improve the contribution to society. |  |  |  |  |  |
| 3 | Transparency in the organization's activity is ensured with respect to its social environment, facilitating communication and cooperation channels with stakeholders. |  |  |  |  |  |
| 4 | An annual report is prepared on the solidarity or social action actions carried out in the organization. |  |  |  |  |  |
| 5 | This report includes the results obtained according to their social or economic importance. |  |  |  |  |  |
|  | **Environmental setting** |  |  |  |  |  |
| 1 | The organization is committed in a public way to prevent the pollution generated by the operations and products, including strategies against climate change, as well as to improve in a continuous way its environmental performance favoring the global objective of sustainable development. |  |  |  |  |  |
| 2 | It identifies, records and evaluates those aspects of the activities developed by the organization, its products and services that cause or may cause impacts to the environment. |  |  |  |  |  |
| 3 | A management program is established with measurable objectives and goals that are consistent with the environmental commitment for the plants or work centers that the company has, with the aim of improving the impacts on the environment produced by its activities. |  |  |  |  |  |
| 4 | The management program (mentioned in 6 c1) is reviewed annually and whenever there are changes in the organization that affect the current identification. |  |  |  |  |  |
| 5 | A risk plan is in place to evaluate, prevent and manage environmental risks associated with the organization's activity, as well as to mitigate adverse impacts on the environment. |  |  |  |  |  |
| 6 | The risk plan includes the records of cases of accidents, incidents and emergency situations, as well as the measures taken for their correction and prevention. |  |  |  |  |  |
| 7 | The risk plan is reviewed annually and whenever there are changes in the organization that may affect the current identification. |  |  |  |  |  |
| 8 | All stakeholders are informed, at least every two years, about environmental aspects associated with the organization's activity. |  |  |  |  |  |
|  | **Investors** |  |  |  |  |  |
| 1 | The guiding principles of transparency, loyalty, and sustainable value creation in relation to the organization are followed. |  |  |  |  |  |
| 2 | A protocol of relations with investors or code of good governance is available in the organization and is also at the disposal of investors. |  |  |  |  |  |
| 3 | The company's annual accounts are made public and accessible. |  |  |  |  |  |
|  | **Competitors** |  |  |  |  |  |
| 1 | The property rights of the company's competitors are respected. |  |  |  |  |  |
| 2 | The use of agreements between the parties or arbitration formulas is encouraged as a way of resolving differences in this regard. |  |  |  |  |  |
| 3 | Improper actions are not used to obtain information about the organization's competitors. |  |  |  |  |  |
| 4 | An updated record is kept that compiles the complaints and requirements made by the competitors. |  |  |  |  |  |
| 5 | No false or biased information is disseminated against the company's competitors. |  |  |  |  |  |
| 6 | The incorporation to associations and forums of common interest is encouraged, which serves as a meeting point with the company's competitors and an exchange of experiences among them. |  |  |  |  |  |
|  | **Public Administrations** |  |  |  |  |  |
| 1 | The opportune communication and dialogue channels are established with the Administrations with which the company is related. |  |  |  |  |  |

**OUR THANKS FOR YOUR COLLABORATION!**
